# Advances in Multifunctional Polymer-Based Nanocomposites

**DOI:** 10.3390/polym16233440

**Published:** 2024-12-08

**Authors:** Jia-Wun Li, Chih-Chia Cheng, Chih-Wei Chiu

**Affiliations:** 1 Department of Materials Science and Engineering, National Taiwan University of Science and Technology, Taipei 10607, Taiwan; 2 Graduate Institute of Applied Science and Technology, National Taiwan University of Science and Technology, Taipei 10607, Taiwan

## 1. Introduction

“Advances in Multifunctional Polymer-Based Nanocomposites” presents the results of pioneering research in a new direction in the field of materials science and engineering technology. This approach synergistically integrates the machinability of polymers [1] with the multifunctional properties of nanomaterials [2] within polymer nanocomposites, yielding composite materials with superior performance. As technology progresses and demands diversify, these nanocomposites will not only enhance material performance but also offer novel application prospects in modern engineering, healthcare, energy, and electronics [3,4,5,6]. These materials exhibit a plethora of exceptional properties, including high mechanical strength, lightweight characteristics, thermal resistance, electrical conductivity, magnetic properties, optoelectronic functionality, gas barrier properties, and dimensional stability. These properties make them critical solutions to transcend the limitations of traditional materials [7].

The distinctive properties of nanocomposites are derived from the elevated specific surface area and quantum size effects of inorganic nanoparticles, which bestow dual advantages in microstructural and macroscopic performance [8]. For example, zero-dimensional (0D) nanoparticles and quantum dots provide precise optoelectronic functionality, one-dimensional (1D) nanorods and nanotubes offer exceptional mechanical properties and electrical conductivity, and two-dimensional (2D) nanomaterials (such as graphene and layered materials) enable superior thermal conductivity, gas barrier properties, and electronic performance [9]. Chemical surface modification and physical mixing can be used to uniformly disperse such nanoparticles within polymer matrices, thereby forming homogeneous composites with superior performances. Nevertheless, attaining optimal material performance necessitates the resolution of challenges pertaining to the dispersion and interfacial behavior of nanoparticles within polymers, a topic that continues to be a primary focus of contemporary research [10]. In recent years, advanced multifunctional polymer-based nanocomposites have demonstrated significant potential across a range of applications, including fuel cells, solar cells, and energy harvesting devices in the energy sector, with the aim of enhancing energy conversion efficiencies [11], as well as in environmental technologies for membrane separation and wastewater treatment, with the objective of achieving efficient filtration and pollutant removal [12]. In the biomedical field, they are employed in drug delivery, biological scaffolds, and tissue engineering to enhance biocompatibility and functionality [13]. They are also utilized in smart devices for wearable sensors, flexible electronics, pressure sensors, and self-healing materials [14] to meet modern technology demands for multifunctionality, lightweight properties, and flexibility. The accelerated advancement of the Internet of Things (IoT) [15], artificial intelligence (AI) [16], and environmental protection [17] has led to a growing need for materials that exhibit both high intelligence and environmental friendliness [18].

It is indubitable that advanced multifunctional polymer-based nanocomposites offer novel solutions to these demands. Moreover, it is anticipated that, in the future, these materials will assume a more pivotal role in the development of innovative products as a result of interdisciplinary research. This Special Issue, entitled *Advances in Multifunctional Polymer-Based Nanocomposites*, will present a comprehensive collection of innovative research and review articles from around the globe. The topics covered will range from fundamental theories to applied explorations.

## 2. Overview of Published Articles

Rajabifar and Rostami [19] (contribution 1) explored the enhancement of the compatibility and mechanical properties of polylactic acid (PLA) and polyolefin elastomer (POE) blends by incorporating various amounts of nanoclay and a fixed amount of nanosilver particles (AgNPs) using a melt blending method. The addition of nanoclay significantly improved the elongation at break (increased to 32.44%) and impact strength (reached 3.46 ± 0.18 kJ/m^2^) of the PLA/POE blend. Furthermore, the nanoclay increased the surface roughness and melt viscosity of the material, restricting polymer chain movement and further enhancing the mechanical properties of the PLA/POE nanocomposites.

Wang and Taheri [20] (contribution 2) compared the impact and post-impact compression properties of 2D and 3D fiber-metal laminates (FMLs). Glass laminate aluminum-reinforced epoxy (GLARE) had better impact resistance than a 3DFML but was thinner and exhibited different failure modes. The compression load capacities of GLARE and 3DFML decreased by 62.6% and 41.5%, respectively. Finite element models accurately predicted the damage evolutions of both materials, offering valuable insights for material life assessment.

Wang et al. [21] (contribution 3) presented the development of a temperature-sensitive electrochemical sensor based on poly(N-isopropylacrylamide) (PNIPAM) and carboxylated multi-walled carbon nanotubes (MWCNTs-COOH) for the reversible detection of dopamine (DA). The sensor was “closed” at low temperatures and “open” at high temperatures due to the shrinkage of the polymer. It showed a broad detection range and low detection limit (193 nM), providing a new avenue for the application of temperature-sensitive polymers.

Alshammari et al. [22] (contribution 4) investigated the integration of g-C_3_N_4_ nanosheets into PVC/PVP polymers to enhance their thermal stability (with a decomposition temperature increase from 262 °C to 276 °C) and optical properties (the refractive index increased to 3.96). As the g-C_3_N_4_ content increased, both the nonlinear refractive index and DC conductivity improved significantly, indicating suitability for optoelectronic sensor applications.

Mohamed et al. [23] (contribution 5) investigated the synthesis of two novel porous polymers (OVS-P-TPA and OVS-P-F) using the Heck coupling reaction. OVS-P-F exhibited a higher thermal stability (444 °C) and specific surface area (375 m^2^/g). OVS-P-TPA demonstrated better performance in photocatalytic hydrogen production (701.9 µmol g^−1^ h^−1^), highlighting its potential for energy storage and catalytic applications.

Echarri-Giacchi and Martín-Martínez [24] (contribution 6) incorporated nano-silica (0.1–0.5 wt%) into aqueous polyurethane to enhance its thermal stability, mechanical properties, and melting enthalpy. However, this also reduced its adhesion and wettability. The optimized dispersion of nano-silica improved the material properties, although excessive content led to a decrease in the final adhesive strength.

Hu et al. [25] (contribution 7) introduced a novel cyclone mixer to enhance the quality of polymer injection molding. After multi-parameter optimization, the cyclone mixer produced fewer defects (9659 ppm vs. 10,688 ppm) and higher quality products compared to traditional screw mixers, making it suitable for injection molding applications.

Sanchez et al. [26] (contribution 8) studied the effect of mixed fillers of AlN and BN on the thermal conductivity of epoxy resin. At a 75 wt% filler loading, the thermal conductivity increased to 10.18 W/m·K, which was 46 times higher than that of pure resin. The material also demonstrated excellent thermal stability and a low thermal expansion coefficient, making it ideal for electronic packaging applications.

Chen et al. [27] (contribution 9) discussed the preparation of cellulose nanofibers (TOCN) by TEMPO oxidation, followed by modification using SI-ATRP technology to create hydrophobic, high-thermal-stability transparent films. These films are suitable for use in biodegradable and renewable biocomposite materials.

Anani et al. [28] (contribution 10) optimized electrospinning parameters to produce spherical calcium alginate microcapsules with diameters of less than 100 µm. Using the response surface method (RSM), the size and sphericity of the microcapsules were precisely predicted and controlled.

Huang et al. [29] (contribution 11) developed a mixed dispersant based on PTT and graphene oxide (GO) to stabilize copper nanoparticles (CuNPs), improving their antioxidant and electrical stability. The material was successfully applied in ECG smart clothing as electrodes for long-term monitoring.

Li et al. [30] (contribution 12) used polymer-assisted dispersants to stabilize platinum nanoparticle (PtNP)/carbon nanotube (CNT) mixtures for use in dye-sensitized solar cells (DSSCs). The material achieved a high conversion efficiency (8.45%) and low cost, offering an effective alternative to traditional platinum counter electrodes.

Alshahri et al. [31] (contribution 13) strengthened LDPE polymers with Bi_2_O_3_ nanofillers to develop a lightweight, non-toxic X-ray shielding material. With the addition of 15 wt% Bi_2_O_3_, the shielding efficiency reached 80%, and the shielding layer thickness was effectively reduced.

Kim et al. [32] (contribution 14) employed surface-modified silica nanoparticles to enhance the thermal stability and mechanical properties of epoxy resin composites. The strong interfacial interaction between the filler and matrix significantly increased the flexural strength, demonstrating potential for commercialization.

A review by Kumar and Chang [33] (contribution 15) examined the unique properties of black phosphorus (BP) and its applications in energy storage, electronics, and biomedicine. To address its environmental stability challenges, the review suggested combining BP with polymers to form composites, potentially paving the way for transitioning from graphene-based products to commercially viable BP applications.

## 3. Conclusions

This Special Issue, entitled *Advances in Multifunctional Polymer-Based Nanocomposites*, provides a comprehensive overview of the latest progress in advanced multifunctional polymer-based nanocomposites. It encompasses a diverse range of explorations, from structural optimization to practical applications. These studies not only demonstrated the substantial improvements in polymer properties resulting from the incorporation of nanofillers but also presented novel strategies for addressing practical challenges through a range of experimental methods and technological advancements. For example, significant advances have been made in the mechanical properties, electrical conductivity, optical characteristics, and thermal stability of these materials, which have the potential to be applied in a number of fields, including energy, environmental protection, biomedicine, and smart devices. It is noteworthy that these studies underscore the potential of a range of nanomaterials and their interactions with polymer matrices. For example, through the rational design of nanoparticle dispersion and interfacial modification methods, synergistic enhancement effects have been achieved at both the macroscopic and microscopic levels. Moreover, many of the studies discussed in this Special Issue have employed advanced fabrication techniques (such as electrospinning, surface modification, and multiscale simulation) to provide effective solutions for overcoming issues such as nanoparticle agglomeration and enhancing dispersion stability. However, it is important to note that while these research achievements are encouraging, there are still challenges and limitations to be addressed. For example, the specific performance requirements in different application scenarios may increase the costs and processing difficulties involved in the fabrication of nanocomposites. Furthermore, the potential environmental and health impacts of nanomaterials necessitate further assessment to ensure their safety in large-scale applications. In the future, as interdisciplinary research deepens and fabrication technologies advance further, advanced multifunctional polymer-based nanocomposites will not only meet the demands of modern society for high-performance and environmentally friendly materials but will also promote the development and practical applications of more innovative products. Concurrently, collaboration between the academic and industrial communities will prove instrumental in reducing costs, enhancing processing performance, and achieving sustainability. These endeavors will facilitate more substantial advancements in modern engineering technologies, environmental protection, biomedicine, and the field of smart devices.

## Data Availability

The data presented in this study are available on request from the corresponding author.
